# Promising *Acinetobacter baumannii* Vaccine Candidates and Drug Targets in Recent Years

**DOI:** 10.3389/fimmu.2022.900509

**Published:** 2022-05-26

**Authors:** Yong Chiang Tan, Chandrajit Lahiri

**Affiliations:** ^1^School of Postgraduate Studies, International Medical University, Kuala Lumpur, Malaysia; ^2^Department of Biological Sciences, Sunway University, Petaling Jaya, Malaysia

**Keywords:** *Acinetobacter baumannii*, vaccine candidate, chimeric vaccine, ghost vaccine, drug target, *In silico* methods, bioinformatics, interactome analysis

## Abstract

In parallel to the uncontrolled use of antibiotics, the emergence of multidrug-resistant bacteria, like *Acinetobacter baumannii*, has posed a severe threat. *A. baumannii* predominates in the nosocomial setting due to its ability to persist in hospitals and survive antibiotic treatment, thereby eventually leading to an increasing prevalence and mortality due to its infection. With the increasing spectra of drug resistance and the incessant collapse of newly discovered antibiotics, new therapeutic countermeasures have been in high demand. Hence, recent research has shown favouritism towards the long-term solution of designing vaccines. Therefore, being a realistic alternative strategy to combat this pathogen, anti-*A. Baumannii* vaccines research has continued unearthing various antigens with variable results over the last decade. Again, other approaches, including pan-genomics, subtractive proteomics, and reverse vaccination strategies, have shown promise for identifying promiscuous core vaccine candidates that resulted in chimeric vaccine constructs. In addition, the integration of basic knowledge of the pathobiology of this drug-resistant bacteria has also facilitated the development of effective multiantigen vaccines. As opposed to the conventional trial-and-error approach, incorporating the *in silico* methods in recent studies, particularly network analysis, has manifested a great promise in unearthing novel vaccine candidates from the *A. baumannii* proteome. Some studies have used multiple *A. baumannii* data sources to build the co-functional networks and analyze them by k-shell decomposition. Additionally, Whole Genomic Protein Interactome (GPIN) analysis has utilized a rational approach for identifying essential proteins and presenting them as vaccines effective enough to combat the deadly pathogenic threats posed by *A. baumannii*. Others have identified multiple immune nodes using network-based centrality measurements for synergistic antigen combinations for different vaccination strategies. Protein-protein interactions have also been inferenced utilizing structural approaches, such as molecular docking and molecular dynamics simulation. Similar workflows and technologies were employed to unveil novel *A. baumannii* drug targets, with a similar trend in the increasing influx of *in silico* techniques. This review integrates the latest knowledge on the development of *A. baumannii* vaccines while highlighting the *in silico* methods as the future of such exploratory research. In parallel, we also briefly summarize recent advancements in *A. baumannii* drug target research.

## Introduction

As one of the dreadful ESKAPE pathogens, *A. baumannii* has become a worldwide threat due to its resistance to a broad spectrum of currently available drugs, especially in the nosocomial setting (1). This is evident from the enormous mortalities of immunocompromised cases (2, 3) by this opportunistic pathogen. For example, a systematic statistical meta-analysis published in The Lancet reported *A. baumannii* as one of the six leading pathogens causing mortalities due to drug resistance, of which carbapenem-resistant *A. baumannii* has caused at least 50,000 deaths globally in the year 2019 (4). Moreover, the healthcare crisis caused by *A. baumannii*, especially the carbapenem-resistant strains, has peaked in intensive care units (ICU) synchronously with the COVID-19 pandemic (5, 6). Therefore, in parallel to the relentless drug resistance acquisition in *A. baumannii* towards currently available antibacterial drugs, biomedical research in discovering novel vaccines and drug targets remains exigent (7, 8).

Novel vaccine and drug development often require decades or even centuries (9, 10). Reportedly, there are no vaccine candidates for A. baumannii that have stepped into clinical trials (11–14). For vaccine candidates to step into clinical studies, assessing their *in vivo* reactogenicity and immunogenicity in animal models becomes mandatory (15). In addition, the pharmacological and toxicological properties of the targeted vaccine candidates are investigated along with the preclinical studies. Additionally, drug target selection, serving as the first and the most crucial step into drug development, is always supported by prior knowledge and robust characterisation of the proteins of interest or their related biological pathways from the scientific literature (16). The preliminary tests of a drug target’s viability include knockout and expression studies of the targeted gene under stress, both for *in vivo* and *in vitro* models (16, 17).

In the light of the rapid development in multi-omic methods, as well as databases curated thereof, the contribution of bioinformatics based studies has been ubiquitous in research efforts on combating multidrug-resistant bacteria (18–21). For instance, by analysing the bacterial genome or proteome *via in silico* approaches such as protein interactome analysis, one can unveil novel crucial proteins in the bacteria of interest, which can potentially be vaccine candidates (22–24). With the increasing entries in experimentally validated 3D structure database, scilicet Protein Data Bank (PDB), the strength of interactions between small molecules and proteins, as well as protein-protein interactions, can now be predicted *via in silico* approaches (25–27). Such a technique has also been employed in vaccine research by computationally inferring peptide-binding onto immune cell receptors (28). Almost similar methods are seen in the *in silico* drug target research. For example, virtual screening of large chemical databases on drug-target of interest can unearth novel antibacterial drugs to be repurposed or further developed (29, 30).

Exploring novel vaccine candidates or streamlining antigenic peptide regions through experimental screening on animal models is tremendously cost and time expensive; therefore, bioinformatics tools in aiding *in silico* vaccine design have been numerous and extensive (31, 32). Linear B- and T-cell epitope prediction tools, such as EpiJen, MHCPred, and NetMHC, have been prevalent and well-recognised as upstream exploratory research in vaccine design (33–35). Moreover, researchers have also developed bioinformatic tools to predict the allergenicity and toxicity of vaccine candidates, such as AllergenFP and ToxinPred, respectively (36, 37).

**Figure 1:**
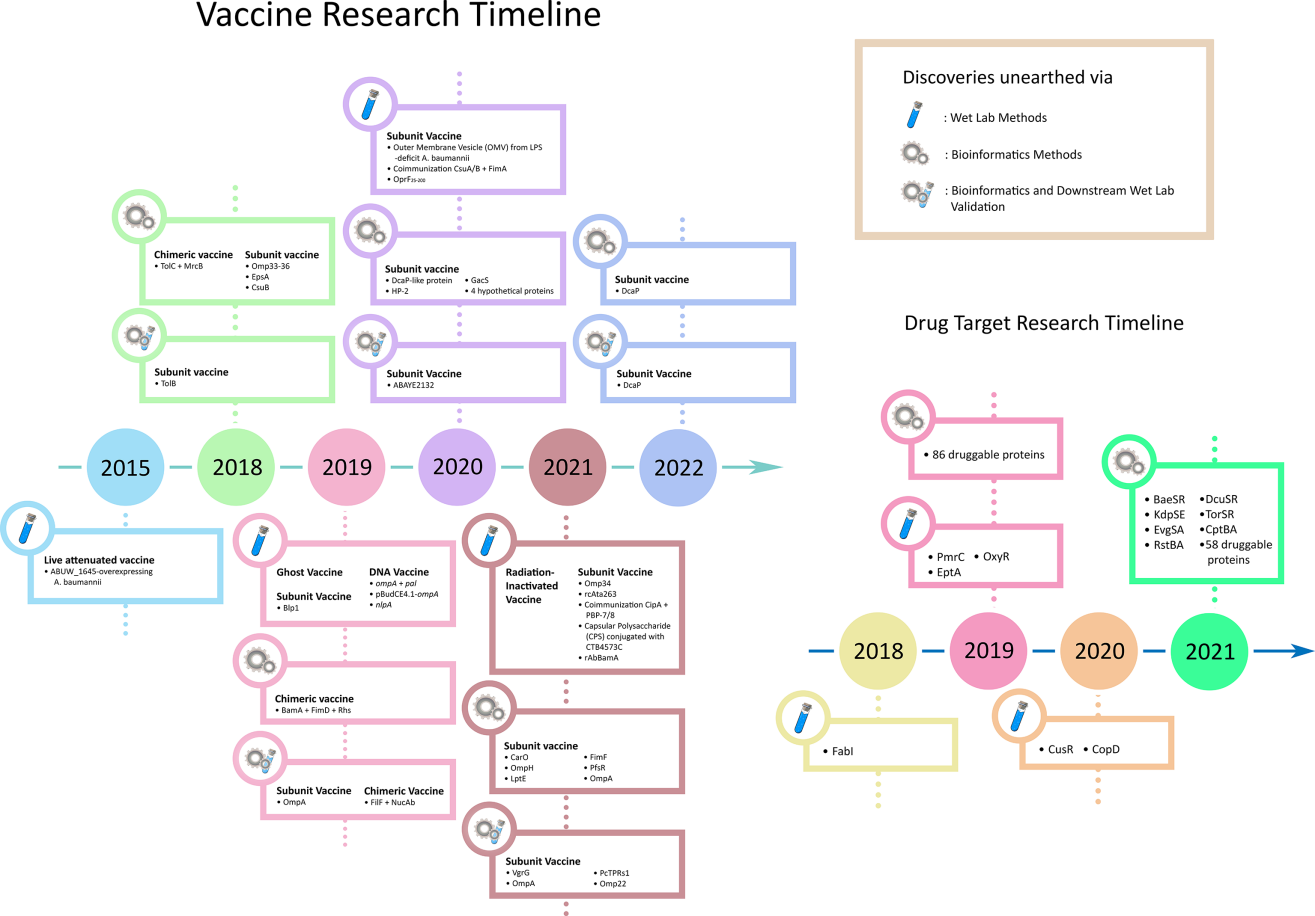
Timeline of the development of vaccines and drug target research of *A. baumannii*.

Over the years, bacteria have gained resistance to the newly developed drugs at a breakneck speed. Consequently, recent exploration favours escalating vaccine research rather than unveiling novel drug targets (38). This review summarizes the recent advancements in *A. baumannii* vaccine design (**Figure 1**). In concordance with the recent studies’ heavy use of computational methods, we accentuate bioinformatics as the future of exploratory research in shepherding drug target selection and vaccine candidature and the multitudinous influx of novel insights into its implication. Moreover, we also briefly discuss *A. baumannii* drug target research, which acts as a temporary coping while we wait for the new vaccines to arrive.

## Inferring Novel Vaccine Candidates From Laboratory Means

Conventional means of inferring novel vaccine candidates require prior established knowledge of the proteins or metabolic pathways of interest and often involve in-depth characterisation of the potential proteins of interest. In addition, deducing new vaccine candidates *via* laboratory means often involves animal models; thus, initial molecular characterisation is almost necessary (39).

### Advancements in Vaccines Derived From Whole-Cell or Anatomical Components

Live vaccines are the most immunologically representative units in establishing vaccination despite posing a danger in regaining infectivity (40). Sequential intraperitoneal immunisation, with sub-lethal doses of different *A. baumannii* strains, has elicited antibody responses towards antigens of multiple strains in CD1 mice (41). Furthermore, a study on virulence switch in *A. baumannii* has highlighted the overexpression of a TetR-type transcriptional regulator, *ABUW_1645*, that facilitated the transformation of the cellular stage from virulent opaque (VIR-O) to avirulent translucent (AV-T) while proposing the potential of *ABUW_1645*-overexpressing *A. baumannii* to be utilized as a live-attenuated vaccine (42).

Bacterial outer membrane vesicle (OMV) has been an attractive vaccine candidate as it contains the outer membrane proteins (43). As lipopolysaccharide (LPS) is abundant in OMV, a study on the efficacy of OMV vaccine on LPS-deficit *A. baumannii* has disproved the effect of LPS on OMV production through electron microscopy (44, 45). In addition, the study also reported similar immunisation strength between OMV isolated from wild-type and purified LPS supplemented LPS-deficit *A. baumannii* by conferring complete protection to the C57BL/6 mouse model (44).

Like inactivated vaccines, bacterial ghosts are gram-negative bacteria depleted of their cytoplasmic and genetic constituents through E-mediated lysis, leaving only the cell envelopes (46). A study on the effectiveness of *A. baumannii* ghosts in conferring vaccinated protections in Sprague-Dawley rats has suggested promising results in terms of effectiveness and safety on various administration methods, including subcutaneous, intramuscular, and intraperitoneal injections (47).

The replicative ability of bacteria can be disabled through the depletion of nucleic acids *via* gamma radiation exposure, with the protection by a specific antioxidant cocktail, and hence eventuating whole-cell inactivated vaccine with better immunogenicity than chemically inactivated vaccines (48–50). Studies showed that intranasal administration of radiation-inactivated *A. baumannii* grown *via* either planktonic cultures in rich media or biofilm cultures in static cultures underneath M9 media had conferred good protection in both healthy and neutropenic mouse models (51).

### Novel Breakthroughs in Subunit Vaccine Research

Due to their safety and stable nature in various conditions, subunit vaccines have been ubiquitous in vaccine design studies (52, 53). Due to the crucial role of Omp34 in enhancing *A. baumannii* virulence and fitness, the Omp34 subunit vaccine has been proposed and reported to be protective in the BALB/c mouse model (54). The 263 amino acid long C-terminal end of an essential *A. baumannii* virulence factor, Ata (rcAta_263_), has elicited immune protection *via* subcutaneous, intraperitoneal, and intranasal challenge in the BALB/c mouse model (55). The combination of two recombinant pilus proteins, namely CsuA/B and FimA, administered *via* subcutaneous injection, has been reported to confer partial (62%) protection in the BALB/c mouse model (56). The subcutaneous co-immunisation of two outer membrane serum resistance factors, CipA and PBP-7/8, has also conferred 80% protection in the C57BL/6 mouse model from the lethal dose *A. baumannii* challenge (39). Moreover, intramuscular injection and passive immunisation of a 711-aa long C-terminal fragment of *A. baumannii* Blp1 protein have protected the lethal challenge in the BALB/c mouse model (57).

As a crucial component on bacterial surfaces, polysaccharides serve as attractive targets for vaccine design. As polysaccharides alone do not elicit an adequate immune response, the conjugation of carrier proteins is often required (58). Upon misuse of antibiotics, *A. baumannii* can produce capsular polysaccharides (CPS) to enhance antibiotic resistance and virulence (59). By introducing the O-glycosylation system from Neisseria into *A. baumannii*, a resulting *in vivo* produced bioconjugated vaccine, having *A. baumannii* CPS and recombinant cholera toxin B subunit (CTB^4573C^) and aluminium hydroxide adjuvant, was reported to manifest good protection and safety in the BALB/c mouse model (60).

By injecting DNA coding for immunogenic antigens into the host *via* a plasmid, immune protection from vaccination can be expected through antigenic expression by host cells (61). Intramuscular injection of DNA vaccine encoding *A. baumannii* OmpA and Pal, adjuvanted by CpG oligodeoxynucleotides (CpG ODN), into C57BL/6 mice exhibited immunological protection against clinical strains of *A. baumannii*, driven by adaptive immune response activation (62). Another study has also designed and proposed the *A. baumannii* OmpA-derived DNA vaccine, pBudCE4.1-*ompA*, as protective against lethal-dose challenge in the BALB/c mouse model (63, 64). Intramuscular injection of *nlpA* DNA vaccine into BALB/c mouse model has also successfully elicited an immune response in a study by Hashemzehi et al. (65). With the recently claimed success of the mRNA vaccine in controlling the coronavirus disease 2019 (COVID-19) pandemic, one can explore the feasibility of the *A. baumannii* mRNA vaccine in the future (66).

Due to the high conservation of specific essential genes across different bacterial species, cross-reactivity of subunit vaccines derived from these genetic sections occurs. For example, a study utilizing the Swiss albino mouse model showed the feasibility of *P. aeruginosa* N-terminal OprF (OprF_25-200_) adjuvanted with Bacillus Calmette-Guerin (BCG) and aluminium hydroxide in conferring cross-reactive immunisation to both *P. aeruginosa* and *A. baumannii* (67). Another study proposed recombinant *A. baumannii* BamA (rAbBamA) protein as a potential vaccine candidate *via* intramuscular challenge on C57/BL6 mouse model adjuvanted by aluminium hydroxide, while reported potential cross-reactivity across K. pneumoniae and E. coli *via* immunoblot assay of anti-rAbBamA (68).

## *In-Silico* Methods in Driving Exploratory Research on Novel Vaccines

With the rapid advancement of bioinformatics in extracting and predicting multi-omic information, as well as the expansion of proteomic and genomic databases, research attempts in unearthing vital proteins as novel vaccine candidates have become meagre cost and increasingly prevalent (69–71). By mapping small protein interactomes (SPIN) of virulent factors, vaccine candidates, and key factors retrieved from literature onto the whole genome protein interactome (GPIN), a study has reported crucial proteins *via* co-functional network analysis and k-shell decomposition, which can serve as potential vaccine candidates (22). Besides, a theoretical study on hypothetical proteins (HPs) in 30 multidrug-resistant *A. baumannii* strains has proposed 4 HPs as potential vaccine candidates *via in silico* immunological analyses (72). In addition, utilizing *in silico* functional analysis, the authors also proposed the druggability of 7 HPs. Upstream subtractive proteomics, combined with linear T and B cell epitopes prediction, structural screening with immune cell receptors, and druggability analysis, have finalised a chimeric subunit vaccine candidate derived from TolC and MrcB in *A. baumannii*, as well as 13 potentially druggable proteins (73).

### Immunoinformatics and Proteomics in Vaccine Design

**Figure 2:**
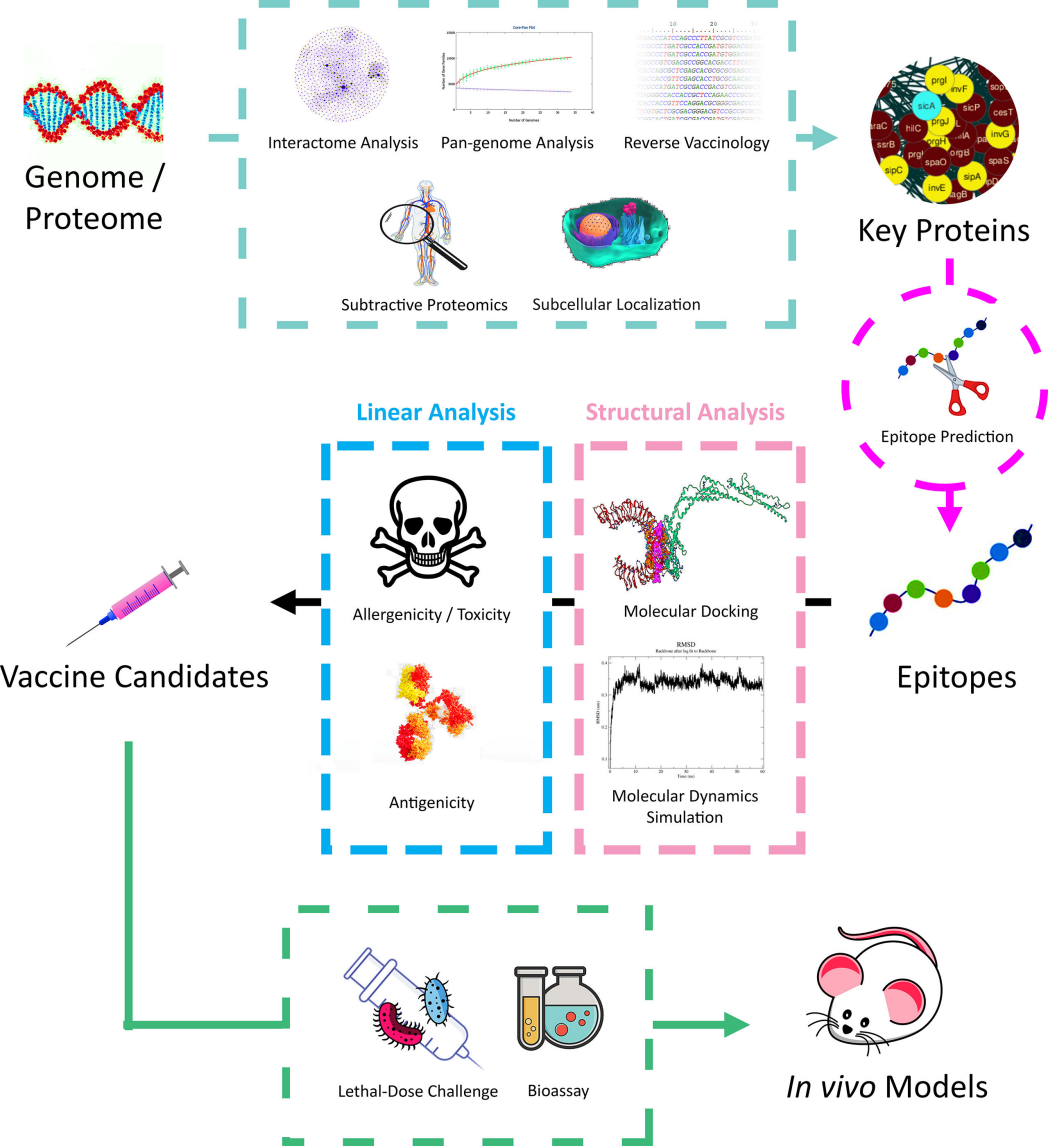
A typical workflow of *in silico* vaccine design in recent studies on *A. baumannii*.

Bioinformatics approaches, especially proteomics, have been extensively applied in recent studies by ushering the mining of core protein candidates in downstream vaccine design and modifications (**Figure 2**). For instance, applying pan genomics in inferring gene conservation across species or strains (74), predicting immunogenicity in proteins and inferring epitopes through reverse vaccinology (75), and inferring essential proteins involved during infection *via* proteomic interactomes (76, 77). Furthermore, predicting effective T and B-cell epitopes has been an essential step during vaccine design in ensuring that only the specific immunogenic parts of the candidate proteins are retained *via* peptide truncation due to cost and time efficiency in laboratory or downstream bulk production (78).

A study has applied reverse vaccinology to 33 genomes of *A. baumannii* strains, and downstream B-cell epitope analysis has indicated the potential of two outer membrane proteins, scilicet a DcaP-like protein and HP-2, a novel hypothetical protein, as vaccine candidates (79). Moreover, Beiranvand et al. has integrated the predicted protein and epitope topology on the bacterial membrane surface to the usual vaccine design workflow, namely B cell epitope, subcellular, antigenicity, and solubility predictions, and finalised with CarO, OmpH, LptE, FimF, and PfsR as top five *A. baumannii* vaccine candidates (80, 81). Furthermore, linear prediction of epitopes and their properties has also been conducted on GacS, a virulence modulator *via* citrate metabolism pathway, resulting in five peptides being proposed as *A. baumannii* vaccine candidates (82).

Genomic and proteomic sequence analyses have been employed to search for the conserved sequence to serve as vaccine candidates. Following transcriptional characterisation of *A. baumannii* Omp33-36 protein to highlight its role during the initial phase of infection, phylogenetic analyses have revealed an 8-aa highly conserved motif (PLAEAAFL), potential enough for vaccine development processing (83). Moreover, comparative genomics on *A. baumannii* OmpA of different strains, with downstream *in silico* prediction of T and B cell epitopes, as well as *in silico* characterisation and molecular docking with TLR-2, has derived a 25 amino acids long vaccine candidate by Sogasu et al. (84). Furthermore, another study has demonstrated the integration of pan genomic analysis of around 4200 genomes, T and B cell epitope predictions, and network-based centrality methods in identifying multiple immunologic nodes in *A. baumannii*, which can result in the elucidation of combinatorial synergy in different antigens for different vaccination strategies (85).

In addition to linear prediction of T and B cell epitopes, structure-based downstream analysis, such as structure modelling, molecular docking, and molecular dynamics (MD) simulation, has been integrated to confer more robust computational insights. For example, utilizing T and B cell epitope prediction, immunological feature screening, molecular docking with immune cell receptors, and downstream integration of *in silico* gene cloning, a multi-epitope vaccine design has been proposed computationally from *A. baumannii* CarO, an outer membrane protein associated with carbapenem resistance (86). Again, utilizing a similar approach, Khalid et al. has also proposed an *in silico* designed vaccine derived from *A. baumannii* DcaP protein (87).

More comprehensive *in silico* approaches have also been conducted in vaccine design. For instance, upstream integration of pan genomics, subtractive proteomics, and reverse vaccinology into T and B cell epitope prediction, pipelined with downstream *in silico* immunological simulation and structure-based screening onto immune cell receptors, two *A. baumannii* multi-epitope vaccine constructs with good safety profiles have been designed (88). Another study on Tigecycline-resistant *A. baumannii* utilized subtractive proteomics, exoproteome and secretome predictions, interactome analysis, as well as other physicochemical and immunological predictions to propose a chimeric vaccine design. The vaccine’s predicted T and B cell epitopes are from three proteins, scilicet BamA, an outer membrane protein assembly complex, FimD, an outer membrane usher protein, and Rhs, a type IV secretion protein (89). An integrated approach of extracting *A. baumannii* virulent factors from the VFDB database, secretome and exoproteome analysis, subtractive proteomics, B and T cell epitope prediction, as well as peptide docking analysis, has resulted in two nine amino acid long potential vaccine candidates derived from EpsA and CsuB, respectively (90).

To facilitate *ex silico* robustness of computationally predicted results, downstream *in vitro* and *in vivo* models have been utilized. For example, predicted T and B cell epitopes of an *A. baumannii* outer membrane protein, TolB, were verified to be antigenic *in vivo*, by utilizing lymphocyte proliferation assay and ELISA, respectively, in a mouse model (91). In another study on the feasibility of vaccine design based on VgrG, a type VI secretion system (T6SS) component, the immunisation of two peptides derived from *A. baumannii* VgrG conserved sequences (*vgrG*_1159-2196_ and *vgrG*_1159-1502_) in BALB/c mice has conferred protection from lethal-dose challenges (92). Again, ABAYE2132, an *A. baumannii* fimbrial protein previously proposed *via* reverse vaccinology, has been translated into an animal study, and its protection capability towards subcutaneously injected BALB/c mouse model was conferred *via* lethal-dose challenge (93). Moreover, a study has proposed a novel synthetic peptide derived from *A. baumannii* OmpA protein using the overlapping regions of predicted T and B cell epitopes, pipelined with three-dose subcutaneous injection in C5BL/6 SPF mouse model, which eventually observed spiked antibody production in the injected mice (94). Another study on the *A. baumannii* OmpA protein has proposed a 27 amino acid long peptide as a vaccine candidate by manifesting protection towards the challenge in C5BL/6 mice (95). Moreover, a recombinant multi-epitope protein has been designed from predicted T and B cell epitopes of *A. baumannii* outer membrane proteins, namely FilF and NucAb, which conferred immunogenicity and protection in the BALB/c mouse model (96). Similar approaches have been adopted by Raoufi et al. on DcaP, a porin protein in *A. baumannii*, which has manifested protection against the challenge in BALB/c mice (97). Furthermore, Abdollahi et al. named a previously hypothetical protein in *A. baumannii*, PcTPRs1, and derived a 101 amino acid long subunit vaccine based on its *in-silico* predicted T and B cell epitopes, subcellular locations, physiochemical properties, as well as *in vivo* challenge on BALB/c mouse model supplemented by Freund’s adjuvant *via* subcutaneous injection (98). Recently, the *A. baumannii* recombinant multi-epitope Omp22 vaccine derived from its predicted T and B cell epitopes, with chitosan and poly lactic-co-glycolic acid (CS-PLGA) nanoparticles encapsulation, has been proposed as a potential nanovaccine candidate with reported protection towards lethal intratracheal challenge on BALB/c mice (99).

### *In Silico* Methodologies to Nominate Vaccine Candidates

With the surfacing of more sophisticated bioinformatics tools over the recent years, in silico approaches in unveiling potential vaccine candidates within the *A. baumannii* proteome have become highly diverse. However, it can be stratified into two major categories: the upstream and the downstream analysis. The upstream analysis of vaccine candidature aims to mine a subset of potential candidates from a vast pool of proteins, usually the whole proteome, genome, and pan-genome. Furthermore, the downstream analysis aims either to shape the protein candidates into effective epitopes or to validate the feasibility of the proposed vaccine candidates, *albeit* computationally.

The upstream analysis of *in silico* vaccine candidature includes protein interactome analysis, which rationally helps to unearth the protein central to the interactome and thus, plays a crucial role in the pathogenesis/pathophysiology of the disease (22). Therein, the connectivity between each protein can be extracted from the protein interaction metadatabase like STRING (100), while network visualisation software tools and plugins like Cytoscape (101), CytoNCA (102), and NetworkAnalyzer (103) help in the analysis. Other analyses, such as *k*-core analysis, can be conducted externally using MATLAB (104). The pan-genome analysis allows the analysis of inter-species or -strain gene conservation, thus eventuating vaccine candidates with a broad spectrum of targets and high tolerance towards mutation. Such analysis can be done *via* the Bacterial Pan Genome Analysis Tool (BPGA) (105) and PanRV pipeline (106). Subtractive proteomics inspects the homology of the target proteome of interest with the host proteome, for example, the *A. baumannii* proteome and human proteome, to avoid integration into the host genome. This can be achieved using the BLASTp tool accelerated with in-house scripts (90). Predictions on the properties of the proteins of interest, such as subcellular localisation of proteins and signal peptides, can be vital in reverse vaccinology. Protein subcellular localisation can be predicted *via* PSORTb (107), CELLO (108), PSLpred (109), and Gneg-mPLoc (110), while the signal peptides localisation can be predicted *via* SignalP (111).

One significant component of the downstream analysis is T and B cell epitope prediction; such bioinformatic tools include ABCPred (112), ProPred (113), as well as IEDB tools such as Discotope (114), ElliPro (115), and TepiTool (116). The predicted epitopes are sometimes backed up by external antigenicity prediction tools, such as Vaxign (117), ANTIGENpro (118), and Vaxijen (119). Safety profiles prediction, such as allergenicity of the predicted epitopes, can be conducted using AllergenFP (36) and AlgPred (120). Besides, ToxinPred (37) gives an idea of the related toxicity. Physiochemical properties such as molecular weight, stability, and hydropathicity, can be predicted using the Expasy ProtParam server (121). The binding capabilities of epitopes to immune cell receptors can also be predicted using the structural method. For instance, the 3D structure of the epitopes can be predicted using I-TASSER (122) and SWISS-MODEL (123). Peptide docking tools such as HADDOCK (124), FireDock (125), and PatchDock (126) can predict epitope binding strength onto immune cell receptors. Eventually, the epitope binding dynamics can be simulated through MD simulation method *via* GROMACS (127).

## Recent Research on Validating Novel Drug Targets

Aside from all the hype on vaccine research, efforts in foraging novel drug targets remain inevitable and in tremendous demand prior to the approval, introduction, and global distribution of new vaccines into the human population.

### Unearthing Drug Targets Using Conventional Means

Like vaccine research, unveiling novel drug targets *via* conventional laboratory approaches requires extensive molecular knowledge or characterisation of the protein of interest or its biological pathway. Most frequently employed approaches in inferring drug targets include knockout study and chemical inactivation of protein function to inspect its essentiality in ensuring bacterial survival (128, 129).

Instead of exploring novel direct bactericidal drug targets, recent research has sought adjuvant therapy drug targets in the molecular components that contribute to bacterial virulence and drug resistance. For instance, colistin, or polymyxin E, has been a prominent final resort for severe *A. baumannii* infection, and its resistance is mediated by the pmrCAB operon, which codes for phosphoethanolamine (PetN) and two-component system (TCS), as well as PetN addition to the LPS lipid A (130). Moreover, a knockout study on colistin resistance has suggested PetN transferases, scilicet PmrC and EptA, as promising drug targets in attenuating colistin resistance in *A. baumannii* while disproving PmrA, a transcriptional regulator mediating PmrC overexpression, as a drug target due to its limited spectrum (131). Furthermore, utilizing a chemical scaffold compound that inhibits the enzymatic activity of FabI, FabI has manifested druggability in *A. baumannii* by enhancing the bactericidal effect of colistin (129).

Aside from attenuating drug resistance, attempts to promote bacterial susceptibility to the immune system have been observed in research studies. Notably, copper has been an interesting antibacterial molecule employed by the immune system to facilitate pathogenic clearance upon infection, manifested by a copper burst in macrophage phagosomes and blood (132–134). In this regard, Williams et al. has reported 11 proteins that result in *A. baumannii* sensitivity to copper upon loss-of-function mutations, with two of them, scilicet CusR and CopD, being validated in the BALB/c mouse model in causing less mortality (135). These proteins rendering copper resistance could serve as potential drug targets in weakening *A. baumannii* virulence. Again, another study has proposed the druggability of OxyR, a transcriptional regulator in hydrogen peroxide detoxification, *via* a knockout study (128). Additionally, through *in vivo* imaging, the authors have supplemented the finding that *A. baumannii* in the lung suffers oxidative stress from hydrogen peroxide.

### *In-Silico* Methods in Mining Novel Drug Targets

**Figure 3:**
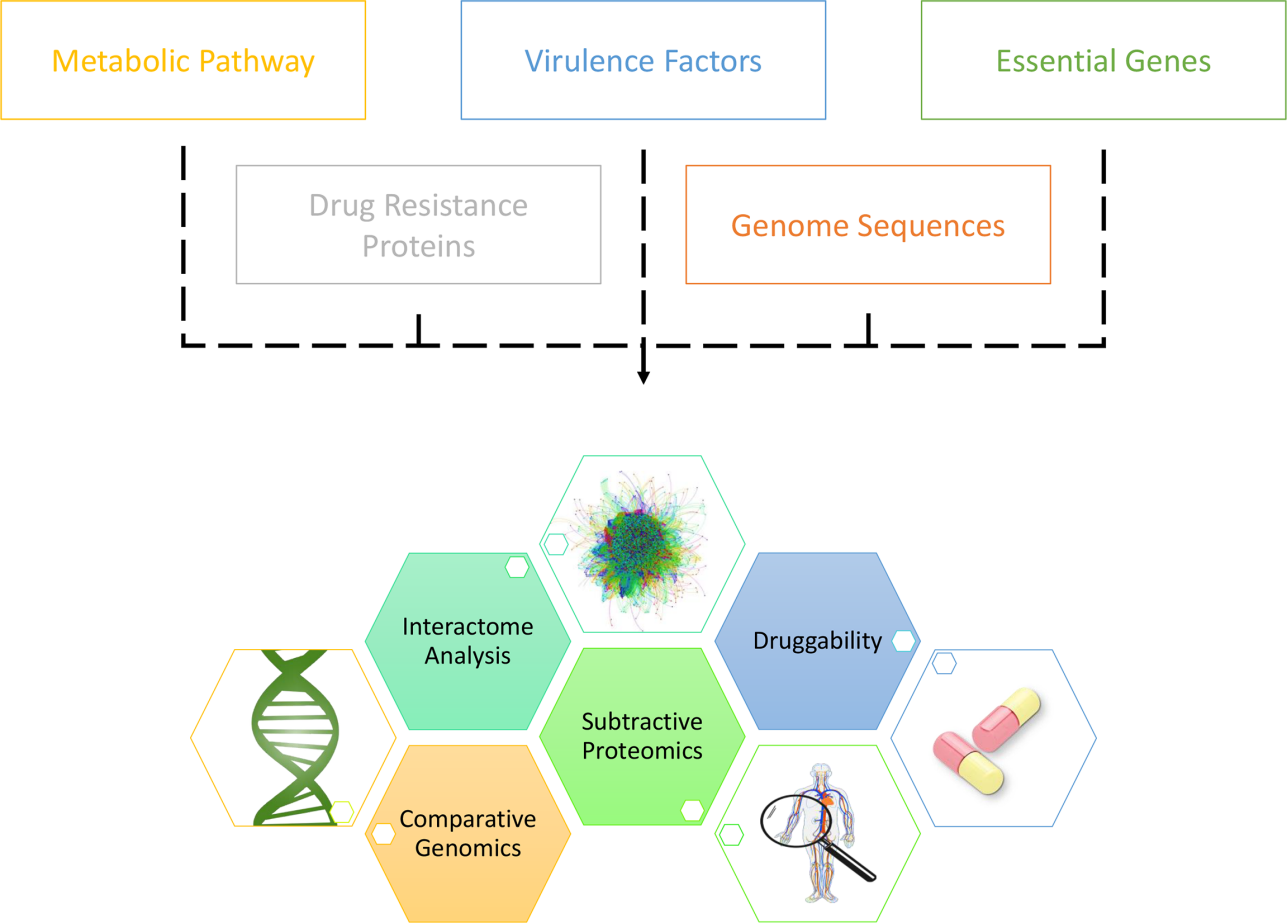
Canonical workflow of unearthing novel drug targets in *A. baumannii* via *the in silico* approaches.

With the enormous growth in the variety and size of biological databases, different databases have been innovatively extracted in inferring drug targets. Potentially bactericidal drug targets burrowed in vast and complex datasets could be unearthed with comprehensive analyses on annotated multi-omic sequences (**Figure 3**) (136). For example, in synchronous to the increasing attention towards the TCS in bacteria, pan-genomics and sequence variation analysis on TCS proteins in seven pathogen species, inclusive of *A. baumannii*, has been conducted by Rajput et al., who have reported BaeSR, KdpDE, EvgSA, RstBA, DcuSR, and TorSR as potentially druggable targets (137). Moreover, the Toxin-Antitoxin database (TADB) pipelined with comparative genomics and phylogenetic analysis, as well as *in vitro* transcriptional analysis upon oxidative and antibiotic stress, has demonstrated the potential druggability of *A. baumannii* CptBA Toxin-Antitoxin system in disrupting intracellular toxin-antitoxin balance, hence eventuating cell suicide (17). Furthermore, integrated analysis of metabolic pathways and chokepoints *via* the KEGG database (138), plasmid proteins, virulence factors *via* the VFDB database (139), and drug resistance proteins *via* the CARD database (140), pipelined with subtractive analysis of essentiality and non-homology, Kaur et al. have presented 58 potentially druggable proteins of which eighteen (18) existed or had their homologs in DrugBank (141, 142). Additionally, utilizing KEGG pathway analysis, essentiality analysis from the DEG database (143), and subtractive proteomics, Uddin et al. have presented eighty-six (86) potentially druggable proteins, with forty-five (45) exhibiting high sequence similarity with the existing drug targets in DrugBank (144).

Besides the above, bioinformatics approaches downstream of drug target identification often include virtual screening *via* molecular docking and MD simulation, and hence, experimentally crystallised protein 3D structures have become a godsend in recent research (29, 30, 145). In this regard, in a study, researchers have crystallised the 3D structures of 29 essential proteins in *A. baumannii* and deposited them in Protein Data Bank (PDB), fifteen (15) of which were recommended to be druggable, based on active site features and sequence homology (146).

## Other Proteomics-Driven Strategies

Aside from the unveiling of novel vaccines and drug targets, recent proteomics research on other strategies such as antimicrobial peptides (AMPs) have been promising in combating *A. baumannii* (147). In a study by Jung et al., *in vivo* screening of AMPs in a mouse model has reported SMAP-29 and TP4 to exhibit prophylactic properties, while dC4 and dN4 exhibited potential therapeutic activity against *A. baumannii* (148). Moreover, a hexahistidine-tagged AMP, namely Lys AB2 P3-His, with a gold nanoparticle carrier, AuNP-Apt, has conferred significant protection to mice against lethal-dose *A. baumannii* challenge (149). Other than direct antimicrobial activities, the additional capabilities of AMPs in enhancing drug effects have been explored. Esc(1-21), a frog-skin AMP derivative, has manifested synergistic membrane-perturbing antibacterial activity with colistin on multidrug-resistant *A. baumannii* clinical isolates (150). *In silico* study on AMPs has been prevalent with the increasing availability of peptide-peptide docking. A virtual screening study by He et al. with a set of cyclic peptides against *A. baumannii* BamA, pipelined with downstream *in vivo* mouse model challenge, has proposed cyclo-RRWWRRW to be membrane-perturbing bactericidal (151). Another study has demonstrated the *in silico* screening of human proteome for encrypted AMPs to be translatable into *in vivo* mouse models (152).

## Conclusion

The increased utilization of bioinformatic approaches in exploring *A. baumannii* vaccines has invited a tremendous influx of passion and novel research outputs in combating the notorious nosocomial pathogen, mainly due to its immense cost and time efficiency. We can unveil potential vaccine candidates by extracting and collating information through different databases with statistical means. In addition to employing bioinformatics tools with straightforward outputs, supplementary approaches, such as comparative genomics, subtractive proteomics, and interactome analyses, have been conducted to strengthen or further streamline the shortlisted vaccine candidates. Similar research trends and methodologies can be observed in unveiling novel drug targets. In the light of the rapid expansion of drug resistance spectra in bacteria, scouring for novel vaccine candidates seemed to be a more attractive choice than tramping in the vicious cycle of bacteria rapidly acquiring drug resistance. Nonetheless, due to the long timeline of vaccine research, unearthing novel drug targets remains crucial while waiting for new vaccines to be developed and distributed. As research progresses, bioinformatics databases and methodologies will continue to confer valuable insights in pushing through the vaccine research to combat the *A. baumannii* infection.

## Author Contributions

YT primarily wrote the manuscript aided by complete editorial up-gradation by CL. In addition, figures were generated by YT with guidance provided by CL. All authors contributed to the article and approved the submitted version.

## Conflict of Interest

The authors declare that the research was conducted in the absence of any commercial or financial relationships that could be construed as a potential conflict of interest.

## Publisher’s Note

All claims expressed in this article are solely those of the authors and do not necessarily represent those of their affiliated organizations, or those of the publisher, the editors and the reviewers. Any product that may be evaluated in this article, or claim that may be made by its manufacturer, is not guaranteed or endorsed by the publisher.

## References

[1] De Oliveira DMP Forde BM Kidd TJ Harris PNA Schembri MA Beatson SA. Antimicrobial Resistance in ESKAPE Pathogens. Clin Microbiol Rev (2020) 33(3):e00181-19. doi: 10.1128/CMR.00181-19 32404435 PMC7227449

[2] Nocera FP Attili A-R De Martino L. Acinetobacter baumannii: Its Clinical Significance in Human and Veterinary Medicine. Pathogens (2021) 10(2):127. doi: 10.3390/pathogens10020127 33513701 PMC7911418

[3] Thompson T. The Staggering Death Toll of Drug-Resistant Bacteria. Nature News, [online]. p. 1. Available at: https://www.nature.com/articles/d41586-022-00228-x [Accessed 10 April 2022]. 10.1038/d41586-022-00228-x 35102288

[4] Murray CJ Ikuta KS Sharara F Swetschinski L Robles Aguilar G Gray A. Antimicrobial Resistance Collaborators. Global Burden of Bacterial Antimicrobial Resistance in 2019: A Systematic Analysis. Lancet (2022) 399(10325):629–55. doi: 10.1016/S0140-6736(21)02724-0 PMC8841637 35065702

[5] Kyriakidis I Vasileiou E Pana ZD Tragiannidis A. Acinetobacter baumannii Antibiotic Resistance Mechanisms. Pathogens (2021) 10(3):373. doi: 10.3390/pathogens10030373 33808905 PMC8003822

[6] Thoma R Seneghini M Seiffert SN Vuichard Gysin D Scanferla G Haller S. The Challenge of Preventing and Containing Outbreaks of Multidrug-Resistant Organisms and Candida Auris During the Coronavirus Disease 2019 Pandemic: Report of a Carbapenem-Resistant Acinetobacter baumannii Outbreak and a Systematic Review of the Literatu. Antimicrob Resist Infect Control (2022) 11(1):12. doi: 10.1186/s13756-022-01052-8 35063032 PMC8777447

[7] Suranadi IW Fatmawati NND Aryabiantara IW Sinardja CD Saputra DJ Senapathi TGA. Acinetobacter baumannii Is an Opportunistic Pathogen as an MDRO Especially on Intensive Ward. Bali J Anesthesiol (2019) 3(2):150–3. doi: 10.15562/bjoa.v3i2.199

[8] Zhang T Xu X Xu C-F Bilya SR Xu W. Mechanical Ventilation-Associated Pneumonia Caused by Acinetobacter baumannii in Northeast China Region: Analysis of Genotype and Drug Resistance of Bacteria and Patients’ Clinical Features Over 7 Years. Antimicrob Resist Infect Control (2021) 10(1):135. doi: 10.1186/s13756-021-01005-7 34526127 PMC8444615

[9] Kim YC Dema B Reyes-Sandoval A. COVID-19 Vaccines: Breaking Record Times to First-in-Human Trials. NPJ Vaccines (2020) 5(1):34. doi: 10.1038/s41541-020-0188-3 32377399 PMC7193619

[10] Matthews H Hanison J Nirmalan N. “Omics”-Informed Drug and Biomarker Discovery: Opportunities, Challenges and Future Perspectives. Proteomes (2016) 4(3):28. doi: 10.3390/proteomes4030028 PMC5217350 28248238

[11] EU Clinical Trials Register. Clinical Trials for Acinetobacter baumannii AND Vaccine (2022). Available at: https://www.clinicaltrialsregister.eu/ctr-search/search?query=Acinetobacter+baumannii+AND+vaccine.

[12] ICTRP. International Clinical Trials Registry Platform (2022). Available at: https://trialsearch.who.int/.

[13] ISRCTN. Acinetobacter baumannii AND ( Interventions: Vaccine ) (2022). Available at: https://www.isrctn.com/search?q=Acinetobacter+baumannii&filters=intervention%3Avaccine&searchType=advanced-search.

[14] NIH. Vaccine | Acinetobacter baumannii (2022). Available at: https://clinicaltrials.gov/ct2/results?cond=Acinetobacter+baumannii&term=vaccine&cntry=&state=&city=&dist.

[15] Leroux-Roels G Bonanni P Tantawichien T Zepp F. Vaccine development. Perspect Vaccinology (2011) 1(1):115–50. doi: 10.1016/j.pervac.2011.05.005

[16] Gashaw I Ellinghaus P Sommer A Asadullah K. What makes a good drug target? Drug Discovery Today (2011) 16(23–24):1037–43. doi: 10.1016/j.drudis.2011.09.007 21945861

[17] ElBanna SA Moneib NA Aziz RK Samir R. Genomics-guided Identification of a Conserved CptBA-like Toxin-antitoxin System in Acinetobacter baumannii. J Adv Res (2021) 30:159–70. doi: 10.1016/j.jare.2020.11.007 PMC8132199 34026293

[18] Behl T Kaur I Sehgal A Singh S Bhatia S Al-Harrasi A. Bioinformatics Accelerates the Major Tetrad: A Real Boost for the Pharmaceutical Industry. Int J Mol Sci (2021) 22(12):6184. doi: 10.3390/ijms22126184 34201152 PMC8227524

[19] Chen B Butte A. Leveraging Big Data to Transform Target Selection and Drug Discovery. Clin Pharmacol Ther (2016) 99(3):285–97. doi: 10.1002/cpt.318 PMC4785018 26659699

[20] Lee K Kim D-W Cha C-J. Overview of Bioinformatic Methods for Analysis of Antibiotic Resistome from Genome and Metagenome Data. J Microbiol (2021) 59(3):270–80. doi: 10.1007/s12275-021-0652-4 33624264

[21] Lv J Deng S Zhang L. A Review of Artificial Intelligence Applications for Antimicrobial Resistance. Biosafety Health (2021) 3(1):22–31. doi: 10.1016/j.bsheal.2020.08.003

[22] Mujawar S Mishra R Pawar S Gatherer D Lahiri C. Delineating the Plausible Molecular Vaccine Candidates and Drug Targets of Multidrug-Resistant Acinetobacter baumannii. Front Cell Infection Microbiol (2019) 9:203. doi: 10.3389/fcimb.2019.00203 PMC6596342 31281799

[23] Pawar S Bramhachari PV Lahiri C. *In Silico* Approaches for Unearthing Bacterial Quorum-Sensing Inhibitors Against Pathogenic Bacteria. In: Implication of Quorum Sensing and Biofilm Formation in Medicine, Agriculture and Food Industry. Singapore: Springer (2019). p. 67–83. doi: 10.1007/978-981-32-9409-7_6.

[24] Swain A Gnanasekar P Prava J Rajeev AC Kesarwani P Lahiri C. A Comparative Genomics Approach for Shortlisting Broad-Spectrum Drug Targets in Nontuberculous Mycobacteria. Microb Drug Resist (2021) 27(2):212–26. doi: 10.1089/mdr.2020.0161 32936741

[25] Burley SK Bhikadiya C Bi C Bittrich S Chen L Crichlow GV. RCSB Protein Data Bank: Powerful New Tools for Exploring 3D Structures of Biological Macromolecules for Basic and Applied Research and Education in Fundamental Biology, Biomedicine, Biotechnology, Bioengineering and Energy Sciences. Nucleic Acids Res (2021) 49(D1):D437–51. doi: 10.1093/nar/gkaa1038 PMC7779003 33211854

[26] Jumper J Evans R Pritzel A Green T Figurnov M Ronneberger O. Highly Accurate Protein Structure Prediction with AlphaFold. Nature (2021) 596(7873):583–9. doi: 10.1038/s41586-021-03819-2 PMC8371605 34265844

[27] Webb B Sali A. Protein Structure Modeling with MODELLER. In: Chen YW Yiu CPB, editors. Structural Genomics. Methods in Molecular Biology, vol 2199. Humana, New York, NY. (2021) p. 239–255. doi: 10.1007/978-1-0716-0892-0_14 33125654

[28] Mahapatra SR Dey J Kaur T Sarangi R Bajoria AA Kushwaha GS. Immunoinformatics and Molecular Docking Studies Reveal a Novel Multi-Epitope Peptide Vaccine Against Pneumonia Infection. Vaccine (2021) 39(42):6221–37. doi: 10.1016/j.vaccine.2021.09.025 34556364

[29] Asghar A Tan Y-C Shahid M Yow Y-Y Lahiri C. Metabolite Profiling of Malaysian Gracilaria edulis Reveals Eplerenone as Novel Antibacterial Compound for Drug Repurposing Against MDR Bacteria. Front Microbiol (2021) 12:653562. doi: 10.3389/fmicb.2021.653562 34276590 PMC8279767

[30] Asghar A Tan YC Zahoor M Zainal Abidin SA Yow Y-Y Khan E. A Scaffolded Approach to Unearth Potential Antibacterial Components from Epicarp of Malaysian Nephelium lappaceum L. Sci Rep (2021) 11(1):13859. doi: 10.1038/s41598-021-92622-0 34226594 PMC8257635

[31] Soleymani S Tavassoli A Housaindokht MR. An Overview of Progress From Empirical to Rational Design in Modern Vaccine Development, With an Emphasis on Computational Tools and Immunoinformatics Approaches. Comput Biol Med (2022) 140:105057. doi: 10.1016/j.compbiomed.2021.105057 34839187

[32] Soria-Guerra RE Nieto-Gomez R Govea-Alonso DO Rosales-Mendoza S. An Overview of Bioinformatics Tools for Epitope Prediction: Implications on Vaccine Development. J Biomed Inf (2015) 53:405–14. doi: 10.1016/j.jbi.2014.11.003 25464113

[33] Doytchinova IA Guan P Flower DR. EpiJen: A Server for Multistep T Cell Epitope Prediction. BMC Bioinf (2006) 7(1):131. doi: 10.1186/1471-2105-7-131 PMC1421443 16533401

[34] Guan P Hattotuwagama CK Doytchinova IA Flower DR. MHCPred 2.0. Appl Bioinf (2006) 5(1):55–61. doi: 10.2165/00822942-200605010-00008 16539539

[35] Lundegaard C Lamberth K Harndahl M Buus S Lund O Nielsen M. NetMHC-3.0: Accurate Web Accessible Predictions of Human, Mouse and Monkey MHC Class I Affinities for Peptides of Length 8–11. Nucleic Acids Res (2008) 36(suppl_2):W509–12. doi: 10.1093/nar/gkn202 PMC2447772 18463140

[36] Dimitrov I Naneva L Doytchinova I Bangov I. AllergenFP: Allergenicity Prediction by Descriptor Fingerprints. Bioinformatics (2014) 30(6):846–51. doi: 10.1093/bioinformatics/btt619 24167156

[37] Gupta S Kapoor P Chaudhary K Gautam A Kumar R Raghava GPS. *In Silico* Approach for Predicting Toxicity of Peptides and Proteins. PloS One (2013) 8(9):e73957. doi: 10.1371/journal.pone.0073957 24058508 PMC3772798

[38] Elshamy AA Aboshanab KM. A Review on Bacterial Resistance to Carbapenems: Epidemiology, Detection and Treatment Options. Future Sci OA (2020) 6(3):FSO438. doi: 10.2144/fsoa-2019-0098 32140243 PMC7050608

[39] Badmasti F Habibi M Firoozeh F Fereshteh S Bolourchi N Goodarzi NN. The Combination of CipA and PBP-7/8 Proteins Contribute to the Survival of C57BL/6 Mice from Sepsis of Acinetobacter baumannii. Microb Pathog (2021) 158:105063. doi: 10.1016/j.micpath.2021.105063 34166729

[40] Minor PD. Live Attenuated Vaccines: Historical Successes and Current Challenges. Virology (2015) 479:379–92. doi: 10.1016/j.virol.2015.03.032 25864107

[41] Kamuyu G Suen Cheng Y Willcocks S Kewcharoenwong C Kiratisin P Taylor PW. Sequential Vaccination With Heterologous Acinetobacter baumannii Strains Induces Broadly Reactive Antibody Responses. Front Immunol (2021) 12:705533. doi: 10.3389/fimmu.2021.705533 34394105 PMC8363311

[42] Chin CY Tipton KA Farokhyfar M Burd EM Weiss DS Rather PN. A High-frequency Phenotypic Switch Links Bacterial Virulence and Environmental Survival in Acinetobacter baumannii. In Nat Microbiol (2018) 3(5):563–569). doi: 10.1038/s41564-018-0151-5 PMC5921939 29693659

[43] Masignani V Pizza M Moxon ER. The Development of a Vaccine Against Meningococcus B Using Reverse Vaccinology. Front Immunol (2019) 10:751. doi: 10.3389/fimmu.2019.00751 31040844 PMC6477034

[44] Pulido MR García-Quintanilla M Pachón J McConnell MJ. A Lipopolysaccharide-Free Outer Membrane Vesicle Vaccine Protects Against Acinetobacter baumannii Infection. Vaccine (2020) 38(4):719–24. doi: 10.1016/j.vaccine.2019.11.043 31843268

[45] Vanaja SK Russo AJ Behl B Banerjee I Yankova M Deshmukh SD. Bacterial Outer Membrane Vesicles Mediate Cytosolic Localisation of LPS and Caspase-11 Activation. Cell (2016) 165(5):1106–19. doi: 10.1016/j.cell.2016.04.015 PMC4874922 27156449

[46] Lubitz P Mayr UB Lubitz W. Applications of Bacterial Ghosts in Biomedicine. In: Guzmán CA Feuerstein GZ (eds) Pharmaceutical Biotechnology. Advances in Experimental Medicine and Biology, vol 655. New York, NY: Springer. (2009) p.159–70. doi: 10.1007/978-1-4419-1132-2_12 20047041

[47] Sheweita SA Batah AM Ghazy AA Hussein A Amara AA. A New Strain of Acinetobacter baumannii and Characterisation of its Ghost as a Candidate Vaccine. J Infection Public Health (2019) 12(6):831–42. doi: 10.1016/j.jiph.2019.05.009 31230953

[48] Datta SK Okamoto S Hayashi T Shin SS Mihajlov I Fermin A. Vaccination with Irradiated Listeria Induces Protective T Cell Immunity. Immunity (2006) 25(1):143–52. doi: 10.1016/j.immuni.2006.05.013 16860763

[49] Gaidamakova EK Myles IA McDaniel DP Fowler CJ Valdez PA Naik S. Preserving Immunogenicity of Lethally Irradiated Viral and Bacterial Vaccine Epitopes Using a Radio- Protective Mn2+-Peptide Complex from Deinococcus. Cell Host Microbe (2012) 12(1):117–24. doi: 10.1016/j.chom.2012.05.011 PMC4073300 22817993

[50] Gayen M Gupta P Morazzani EM Gaidamakova EK Knollmann-Ritschel B Daly MJ. Deinococcus Mn2+-Peptide Complex: A Novel Approach to Alphavirus Vaccine Development. Vaccine (2017) 35(29):3672–81. doi: 10.1016/j.vaccine.2017.05.016 28576570

[51] Dollery SJ Zurawski DV Gaidamakova EK Matrosova VY Tobin JK Wiggins TJ. Radiation-Inactivated Acinetobacter baumannii Vaccine Candidates. Vaccines (2021) 9(2), 96. doi: 10.3390/vaccines9020096 33514059 PMC7912630

[52] Baxter D. Active and Passive Immunity, Vaccine Types, Excipients and Licensing. Occup Med (2007) 57(8):552–6. doi: 10.1093/occmed/kqm110 18045976

[53] Vartak A Sucheck S. Recent Advances in Subunit Vaccine Carriers. Vaccines (2016) 4(2):12. doi: 10.3390/vaccines4020012 PMC4931629 27104575

[54] Naghipour Erami A Rasooli I Jahangiri A Darvish Alipour Astaneh S. Anti-Omp34 Antibodies Protect Against Acinetobacter baumannii in a Murine Sepsis Model. Microbial Pathogenesis (2021) 161(Pt B):105291. doi: 10.1016/j.micpath.2021.105291 34798280

[55] Hatefi Oskuei R Darvish Alipour Astaneh S Rasooli I. A Conserved Region of Acinetobacter Trimeric Autotransporter Adhesion, Ata, Provokes Suppression of Acinetobacter baumannii Virulence. Arch Microbiol (2021) 203(6):3483–93. doi: 10.1007/s00203-021-02343-1 33907866

[56] Ramezanalizadeh F Owlia P Rasooli I. Type I Pili, CsuA/B and FimA Induce a Protective Immune Response Against Acinetobacter baumannii. Vaccine (2020) 38(34):5436–46. doi: 10.1016/j.vaccine.2020.06.052 32600914

[57] Skerniškytė J Karazijaitė E Deschamps J Krasauskas R Armalytė J Briandet R. Blp1 Protein Shows Virulence-Associated Features and Elicits Protective Immunity to Acinetobacter baumannii Infection. BMC Microbiol (2019) 19(1):259. doi: 10.1186/s12866-019-1615-3 31752683 PMC6873735

[58] Harding CM Feldman MF. Glycoengineering Bioconjugate Vaccines, Therapeutics, and Diagnostics in E. Coli. Glycobiology (2019) 29(7):519–29. doi: 10.1093/glycob/cwz031 PMC6583762 30989179

[59] Geisinger E Isberg RR. Antibiotic Modulation of Capsular Exopolysaccharide and Virulence in Acinetobacter baumannii. PloS Pathog (2015) 11(2):e1004691. doi: 10.1371/journal.ppat.1004691 25679516 PMC4334535

[60] Li X Pan C Liu Z Sun P Hua X Feng E. Safety and Immunogenicity of A New Glycoengineered Vaccine Against Acinetobacter baumannii in Mice. Microbial Biotechnology (2021) 15(2):703–16. doi: 10.1111/1751-7915.13770 PMC8867989 33755314

[61] Grodeland G Fredriksen AB Løset GÅ. Vikse E Fugger L Bogen B. Antigen Targeting to Human HLA Class II Molecules Increases Efficacy of DNA Vaccination. J Immunol (2016) 197(9):3575–85. doi: 10.4049/jimmunol.1600893 PMC5073356 27671110

[62] Lei L Yang F Zou J Jing H Zhang J Xu W. DNA Vaccine Encoding OmpA and Pal from Acinetobacter baumannii Efficiently Protects Mice Against Pulmonary Infection. Mol Biol Rep (2019) 46(5):5397–408. doi: 10.1007/s11033-019-04994-2 31342294

[63] Ansari H Doosti A Kargar M Bijanzadeh M Jaafarinia M. Cloning of ompA Gene from Acinetobacter baumannii into the Eukaryotic Expression Vector pBudCE4.1 as DNA Vaccine. Indian J Microbiol (2018) 58(2):174–81. doi: 10.1007/s12088-017-0705-x PMC5891471 29651176

[64] Ansari H Tahmasebi-Birgani M Bijanzadeh M Doosti A Kargar M. Study of the Immunogenicity of Outer Membrane Protein A (ompA) Gene from Acinetobacter baumannii as DNA Vaccine Candidate *in vivo* . Iranian J Basic Med Sci (2019) 22(6):669–75. doi: 10.22038/ijbms.2019.30799.7427 PMC6570755 31231495

[65] Hashemzehi R Doosti A Kargar M Jaafarinia M. Cloning and Expression of nlpA Gene as DNA Vaccine Candidate Against Acinetobacter baumannii. Mol Biol Rep (2018) 45(4):395–401. doi: 10.1007/s11033-018-4167-y 29790084

[66] van Gils MJ van Willigen HDG Wynberg E Han AX van der Straten K Burger JA. A Single mRNA Vaccine Dose in COVID-19 Patients Boosts Neutralising Antibodies Against SARS-CoV-2 and Variants of Concern. Cell Rep Med (2022) 3(1):100486. doi: 10.1016/j.xcrm.2021.100486 35103254 PMC8668345

[67] Bahey-El-Din M Mohamed SA Sheweita SA Haroun M Zaghloul TI. Recombinant N‐Terminal Outer Membrane Porin (OprF) of Pseudomonas Aeruginosa is a Promising Vaccine Candidate Against Both P. Aeruginosa and Some Strains of Acinetobacter baumannii. Int J Med Microbiol IJMM (2020) 310(3):151415. doi: 10.1016/j.ijmm.2020.151415 32156509

[68] Vieira de Araujo AE Conde LV da Silva Junior HC de Almeida Machado L Lara FA Chapeaurouge A. Cross-Reactivity and Immunotherapeutic Potential of BamA Recombinant Protein From Acinetobacter baumannii. Microbes Infect (2021) 23(4–5):104801. doi: 10.1016/j.micinf.2021.104801 33582283

[69] Borry P Bentzen HB Budin-Ljøsne I Cornel MC Howard HC Feeney O. The Challenges of the Expanded Availability of Genomic Information: An Agenda-Setting Paper. J Commun Genet (2018) 9(2):103–16. doi: 10.1007/s12687-017-0331-7 PMC5849701 28952070

[70] Lahiri C Pawar S Sabarinathan R Ashraf MI Chand Y Chakravortty D. Interactome Analyses of Salmonella Pathogenicity Islands Reveal SicA Indispensable for Virulence. J Theor Biol (2014) 363:188–97. doi: 10.1016/j.jtbi.2014.08.013 25128737

[71] Phillips KA Douglas MP Marshall DA. Expanding Use of Clinical Genome Sequencing and the Need for More Data on Implementation. JAMA (2020) 324(20):2029. doi: 10.1001/jama.2020.19933 33104159 PMC7686292

[72] Zadeh Hosseingholi E Zarrini G Pashazadeh M Gheibi Hayat SM Molavi G. *In Silico* Identification of Probable Drug and Vaccine Candidates Against Antibiotic-Resistant Acinetobacter baumannii. Microb Drug Resist (Larchmont NY) (2020) 26(5):456–67. doi: 10.1089/mdr.2019.0236 31742478

[73] Solanki V Tiwari V. Subtractive Proteomics to Identify Novel Drug Targets and Reverse Vaccinology for the Development of Chimeric Vaccine Against Acinetobacter baumannii. Sci Rep (2018) 8(1):9044. doi: 10.1038/s41598-018-26689-7 29899345 PMC5997985

[74] Tettelin H Masignani V Cieslewicz MJ Donati C Medini D Ward NL. Genome Analysis of Multiple Pathogenic Isolates of Streptococcus Agalactiae: Implications for the Microbial “Pan-Genome”. Proc Natl Acad Sci (2005) 102(39):13950–5. doi: 10.1073/pnas.0506758102 PMC1216834 16172379

[75] Vivona S Gardy JL Ramachandran S Brinkman FSL Raghava GPS Flower DR. Computer-Aided Biotechnology: From Immuno-Informatics to Reverse Vaccinology. Trends Biotechnol (2008) 26(4):190–200. doi: 10.1016/j.tibtech.2007.12.006 18291542

[76] Mishra R Tan YC El-Aal AAAA Lahiri C. Computational Identification of the Plausible Molecular Vaccine Candidates of Multidrug-Resistant Salmonella Enterica. In: Lamas A Regal P Franco CM (Eds.), Salmonella Spp. - A Global Challenge. IntechOpen (2021). doi: 10.5772/intechopen.95856

[77] Pasala C Chilamakuri CSR Katari SK Nalamolu RM Bitla AR Umamaheswari A. An *in Silico* Study: Novel Targets for Potential Drug and Vaccine Design Against Drug Resistant H. Pylori. Microbial Pathogenesis. (2018) 122:156–61. doi: 10.1016/j.micpath.2018.05.037 29800696

[78] Raoufi E Hemmati M Eftekhari S Khaksaran K Mahmodi Z Farajollahi MM. Epitope Prediction by Novel Immunoinformatics Approach: A State-Of-the-Art Review. Int J Pept Res Ther (2020) 26(2):1155–63. doi: 10.1007/s10989-019-09918-z PMC7224030 32435171

[79] Fereshteh S Abdoli S Shahcheraghi F Ajdary S Nazari M Badmasti F. New Putative Vaccine Candidates Against Acinetobacter baumannii Using the Reverse Vaccinology Method. Microb Pathog (2020) 143:104114. doi: 10.1016/j.micpath.2020.104114 32145321

[80] Bagos PG Liakopoulos TD Spyropoulos IC Hamodrakas SJ. PRED-TMBB: A Web Server for Predicting the Topology of -Barrel Outer Membrane Proteins. Nucleic Acids Res (2004) 32(Web Server):W400–4. doi: 10.1093/nar/gkh417 PMC441555 15215419

[81] Beiranvand S Doosti A Mirzaei SA. Putative Novel B-Cell Vaccine Candidates Identified by Reverse Vaccinology and Genomics Approaches to Control Acinetobacter baumannii Serotypes. Infect Genet Evol J Mol Epidemiol Evolution Genet Infect Dis (2021) 96:105138. doi: 10.1016/j.meegid.2021.105138 34793968

[82] Smiline Girija AS. Delineating the Immuno-Dominant Antigenic Vaccine Peptides Against gacS-Sensor Kinase in Acinetobacter baumannii: An *in Silico* Investigational Approach. Front Microbiol (2020) 11:2078. doi: 10.3389/fmicb.2020.02078 33013757 PMC7506167

[83] Novović K Mihajlović S Dinić M Malešević M Miljković M Kojić M. Acinetobacter Spp. Porin Omp33-36: Classification and Transcriptional Response to Carbapenems and Host Cells. PloS One (2018) 13(8):e0201608. doi: 10.1371/journal.pone.0201608 30071077 PMC6072067

[84] Sogasu D Girija ASS Gunasekaran S Priyadharsini JV. Molecular Characterisation and Epitope-Based Vaccine Predictions for ompA Gene Associated With Biofilm Formation in Multidrug-Resistant Strains of A.baumannii. In Silico Pharmacol (2021) 9(1):15. doi: 10.1007/s40203-020-00074-7 33520594 PMC7829033

[85] McConnell MJ Martín-Galiano AJ. Designing Multi-Antigen Vaccines Against Acinetobacter baumannii Using Systemic Approaches. Frontiers in Immunology. (2021) 12:1223. doi: 10.3389/fimmu.2021.666742 PMC8085427 33936107

[86] Touhidinia M Sefid F Bidakhavidi M. Design of a Multi-Epitope Vaccine Against Acinetobacter baumannii Using Immunoinformatics Approach. Int J Pept Res Ther (2021) 27(4):2417–37. doi: 10.1007/s10989-021-10262-4 PMC8397861 34483787

[87] Khalid K Irum S Ullah SR Andleeb S. *In-Silico* Vaccine Design Based on a Novel Vaccine Candidate Against Infections Caused by Acinetobacter baumannii. Int J Pept Res Ther (2022) 28(1):16. doi: 10.1007/s10989-021-10316-7 34873398 PMC8636788

[88] Shahid F Zaheer T Ashraf ST Shehroz M Anwer F Naz A. Chimeric Vaccine Designs Against Acinetobacter baumannii Using Pan Genome and Reverse Vaccinology Approaches. Sci Rep (2021) 11(1):13213. doi: 10.1038/s41598-021-92501-8 34168196 PMC8225639

[89] Ahmad S Ranaghan KE Azam SS. Combating Tigecycline Resistant Acinetobacter baumannii: A Leap Forward Towards Multi-Epitope Based Vaccine Discovery. Eur J Pharm Sci: Off J Eur Fed Pharm Sci (2019) 132:1–17. doi: 10.1016/j.ejps.2019.02.023 30797936

[90] Ahmad S Azam SS. A Novel Approach of Virulome Based Reverse Vaccinology for Exploring and Validating Peptide-Based Vaccine Candidates Against the Most Troublesome Nosocomial Pathogen: Acinetobacter baumannii. J Mol Graph Model (2018) 83:1–11. doi: 10.1016/j.jmgm.2018.04.020 29753164

[91] Song X Zhang H Zhang D Xie W Zhao G. Bioinformatics Analysis and Epitope Screening of a Potential Vaccine Antigen TolB From Acinetobacter baumannii Outer Membrane Protein. Infect Genet Evol: J Mol Epidemiol Evolution Genet Infect Dis (2018) 62:73–9. doi: 10.1016/j.meegid.2018.04.019 29673984

[92] Pazoki M Darvish Alipour Astaneh S Ramezanalizadeh F Jahangiri A Rasooli I. Immunoprotectivity of Valine-Glycine Repeat Protein G, a Potent Mediator of Pathogenicity, Against Acinetobacter baumannii. Mol Immunol (2021) 135:276–84. doi: 10.1016/j.molimm.2021.04.026 33940514

[93] Mahmoudi Z Rasooli I Jahangiri A Darvish Alipour Astaneh S. Prevention of Nosocomial Acinetobacter baumannii Infections With a Conserved Immunogenic Fimbrial Protein. APMIS: Acta Patholog Microbiolog Immunolog Scand (2020) 128(7):476–83. doi: 10.1111/apm.13061 32445596

[94] Mehdinejadiani K Bandehpour M Hashemi A Ranjbar MM Taheri S Jalali SA. *In Silico* Design and Evaluation of Acinetobacter baumannii Outer Membrane Protein a Antigenic Peptides As Vaccine Candidate in Immunised Mice. Iranian J Allergy Asthma Immunol (2019) 18(6):655–63. doi: 10.18502/ijaai.v18i6.2178 32245309

[95] Mehdinejadiani K Hashemi A Bandehpour M Rahmani H Ranjbar MM Yardel V. Evaluationof the New Outer Membrane Protein A Epitope-Based Vaccines for Mice Model of Acinetobacter baumannii Associated Pneumonia and Sepsis Infection. Iranian J Allergy Asthma Immunol (2021) 20(5):537–49. doi: 10.18502/ijaai.v20i5.7404 34664813

[96] Ren S Guan L Dong Y Wang C Feng L Xie Y. Design and Evaluation of a Multi-Epitope Assembly Peptide Vaccine Against Acinetobacter baumannii Infection in Mice. Swiss Med Wkly (2019) 149:w20052. doi: 10.4414/smw.2019.20052 31203576

[97] Raoufi Z Abdollahi S Armand R. DcaP Porin and its Epitope-Based Subunit Promise Effective Vaccines Against Acinetobacter baumannii; *in-Silico* and *in-Vivo* Approaches. Microb Pathog (2022) 162:105346. doi: 10.1016/j.micpath.2021.105346 34864145

[98] Abdollahi S Raoufi Z Fakoor MH. Physicochemical and Structural Characterisation, Epitope Mapping and Vaccine Potential Investigation of a New Protein Containing Tetratrico Peptide Repeats of Acinetobacter baumannii: An In-Silico and *In-vivo* Approach. *Mol Immunol* . (2021) 140:22–34. doi: 10.1016/j.molimm.2021.10.004 34649027

[99] Du X Xue J Jiang M Lin S Huang Y Deng K. A Multiepitope Peptide, rOmp22, Encapsulated in Chitosan-PLGA Nanoparticles as a Candidate Vaccine Against Acinetobacter baumannii Infection. Int J Nanomed (2021) 16:1819–36. doi: 10.2147/IJN.S296527 PMC7942956 33707942

[100] Szklarczyk D Gable AL Nastou KC Lyon D Kirsch R Pyysalo S. The STRING Database in 2021: Customisable Protein–Protein Networks, and Functional Characterisation of User-Uploaded Gene/Measurement Sets. Nucleic Acids Res (2021) 49(D1):D605–12. doi: 10.1093/nar/gkaa1074 PMC7779004 33237311

[101] Shannon P Markiel A Ozier O Baliga NS Wang JT Ramage D. Cytoscape: A Software Environment for Integrated Models of Biomolecular Interaction Networks. Genome Res (2003) 13(11):2498–504. doi: 10.1101/gr.1239303 PMC403769 14597658

[102] Tang Y Li M Wang J Pan Y Wu F-X. CytoNCA: A Cytoscape Plugin for Centrality Analysis and Evaluation of Protein Interaction Networks. Biosystems (2015) 127:67–72. doi: 10.1016/j.biosystems.2014.11.005 25451770

[103] Assenov Y Ramírez F Schelhorn S-E Lengauer T Albrecht M. Computing Topological Parameters of Biological Networks. Bioinformatics (2008) 24(2):282–4. doi: 10.1093/bioinformatics/btm554 18006545

[104] Ashraf MI Ong S-K Mujawar S Pawar S More P Paul S. A Side-Effect Free Method for Identifying Cancer Drug Targets. Sci Rep (2018) 8(1):6669. doi: 10.1038/s41598-018-25042-2 29703908 PMC5923273

[105] Chaudhari NM Gupta VK Dutta C. BPGA- an Ultra-Fast Pan-Genome Analysis Pipeline. Sci Rep (2016) 6(1):24373. doi: 10.1038/srep24373 27071527 PMC4829868

[106] Naz K Naz A Ashraf ST Rizwan M Ahmad J Baumbach J. PanRV: Pangenome-Reverse Vaccinology Approach for Identifications of Potential Vaccine Candidates in Microbial Pangenome. BMC Bioinf (2019) 20(1):123. doi: 10.1186/s12859-019-2713-9 PMC6419457 30871454

[107] Yu NY Wagner JR Laird MR Melli G Rey S Lo R. PSORTb 3.0: Improved Protein Subcellular Localisation Prediction With Refined Localisation Subcategories and Predictive Capabilities for All Prokaryotes. Bioinformatics (2010) 26(13):1608–15. doi: 10.1093/bioinformatics/btq249 PMC2887053 20472543

[108] Yu C-S Lin C-J Hwang J-K. Predicting Subcellular Localisation of Proteins for Gram-Negative Bacteria by Support Vector Machines Based on N -Peptide Compositions. Protein Sci (2004) 13(5):1402–6. doi: 10.1110/ps.03479604 PMC2286765 15096640

[109] Bhasin M Garg A Raghava GPS. PSLpred: Prediction of Subcellular Localisation of Bacterial Proteins. Bioinformatics (2005) 21(10):2522–4. doi: 10.1093/bioinformatics/bti309 15699023

[110] Shen H-B Chou K-C. Gneg-Mploc: A Top-Down Strategy to Enhance the Quality of Predicting Subcellular Localisation of Gram-Negative Bacterial Proteins. J Theor Biol (2010) 264(2):326–33. doi: 10.1016/j.jtbi.2010.01.018 20093124

[111] Almagro Armenteros JJ Tsirigos KD Sønderby CK Petersen TN Winther O Brunak S. SignalP 5.0 Improves Signal Peptide Predictions Using Deep Neural Networks. Nat Biotechnol (2019) 37(4):420–3. doi: 10.1038/s41587-019-0036-z 30778233

[112] Saha S Raghava GPS. Prediction of Continuous B-Cell Epitopes in an Antigen Using Recurrent Neural Network. Proteins: Structure Function Bioinf (2006) 65(1):40–8. doi: 10.1002/prot.21078 16894596

[113] Singh H Raghava GPS. ProPred: Prediction of HLA-DR Binding Sites. Bioinformatics (2001) 17(12):1236–7. doi: 10.1093/bioinformatics/17.12.1236 11751237

[114] Haste Andersen P Nielsen M Lund O. Prediction of Residues in Discontinuous B-Cell Epitopes Using Protein 3D Structures. Protein Sci (2006) 15(11):2558–67. doi: 10.1110/ps.062405906 PMC2242418 17001032

[115] Ponomarenko J Bui H-H Li W Fusseder N Bourne PE Sette A. ElliPro: A New Structure-Based Tool for the Prediction of Antibody Epitopes. BMC Bioinf (2008) 9(1):514. doi: 10.1186/1471-2105-9-514 PMC2607291 19055730

[116] Paul S Sidney J Sette A Peters B. TepiTool: A Pipeline for Computational Prediction of T Cell Epitope Candidates. Curr Protoc Immunol (2016) 114(1):18–19. doi: 10.1002/cpim.12 27479659 PMC4981331

[117] He Y Xiang Z Mobley HLT. Vaxign: The First Web-Based Vaccine Design Program for Reverse Vaccinology and Applications for Vaccine Development. J of Biomed and Biotechnol. (2010) 2010:1–15. doi: 10.1155/2010/297505 PMC2910479 20671958

[118] Magnan CN Zeller M Kayala MA Vigil A Randall A Felgner PL. High-Throughput Prediction of Protein Antigenicity Using Protein Microarray Data. Bioinformatics (2010) 26(23):2936–43. doi: 10.1093/bioinformatics/btq551 PMC2982151 20934990

[119] Doytchinova IA Flower DR. VaxiJen: A Server for Prediction of Protective Antigens, Tumour Antigens and Subunit Vaccines. BMC Bioinf (2007) 8(1):4. doi: 10.1186/1471-2105-8-4 PMC1780059 17207271

[120] Sharma N Patiyal S Dhall A Pande A Arora C Raghava GP. AlgPred 2.0: An Improved Method for Predicting Allergenic Proteins and Mapping of IgE Epitopes. Briefings Bioinf (2021) 22(4):bbaa294. doi: 10.1093/bib/bbaa294 33201237

[121] Gasteiger E Hoogland C Gattiker A Duvaud S Wilkins MR Appel RD. Protein Identification and Analysis Tools on the ExPASy Server BT - . The Proteomics Protocols Handbook (2005) (Walker JM (ed.) p.571–607). Humana Press. 10.1385/1-59259-890-0:571

[122] Yang J Zhang Y. I-TASSER Server: New Development for Protein Structure and Function Predictions. Nucleic Acids Res (2015) 43(W1):W174–81. doi: 10.1093/nar/gkv342 PMC4489253 25883148

[123] Waterhouse A Bertoni M Bienert S Studer G Tauriello G Gumienny R. SWISS-MODEL: Homology Modelling of Protein Structures and Complexes. Nucleic Acids Res (2018) 46(W1):W296–303. doi: 10.1093/nar/gky427 PMC6030848 29788355

[124] Dominguez C Boelens R Bonvin AMJJ. HADDOCK: A Protein–Protein Docking Approach Based on Biochemical or Biophysical Information. J Am Chem Soc (2003) 125(7):1731–7. doi: 10.1021/ja026939x 12580598

[125] Mashiach E Schneidman-Duhovny D Andrusier N Nussinov R Wolfson HJ. FireDock: A Web Server for Fast Interaction Refinement in Molecular Docking. Nucleic Acids Res (2008) 36(Web Server):W229–32. doi: 10.1093/nar/gkn186 PMC2447790 18424796

[126] Schneidman-Duhovny D Inbar Y Nussinov R Wolfson HJ. PatchDock and SymmDock: Servers for Rigid and Symmetric Docking. Nucleic Acids Res (2005) 33(Web Server):W363–7. doi: 10.1093/nar/gki481 PMC1160241 15980490

[127] Abraham MJ Murtola T Schulz R Páll S Smith JC Hess B. GROMACS: High Performance Molecular Simulations Through Multi-Level Parallelism from Laptops to Supercomputers. SoftwareX (2015) 1–2, 19–25. doi: 10.1016/j.softx.2015.06.001

[128] Juttukonda LJ Green ER Lonergan ZR Heffern MC Chang CJ Skaar EP. Acinetobacter baumannii OxyR Regulates the Transcriptional Response to Hydrogen Peroxide. Infect Immun (2019) 87(1):e00413-18. doi: 10.1128/IAI.00413-18 30297527 PMC6300632

[129] Kwon J Mistry T Ren J Johnson ME Mehboob S. A Novel Series of Enoyl Reductase Inhibitors Targeting the ESKAPE pathogens, Staphylococcus aureus and Acinetobacter baumannii. Bioorg Med Chem (2018) 26(1):65–76. doi: 10.1016/j.bmc.2017.11.018 29162308 PMC5733704

[130] Olaitan AO Morand S Rolain J-M. Mechanisms of Polymyxin Resistance: Acquired and Intrinsic Resistance in Bacteria. Front Microbiol (2014) 5:643. doi: 10.3389/fmicb.2014.00643 25505462 PMC4244539

[131] Trebosc V Gartenmann S Tötzl M Lucchini V Schellhorn B Pieren M. Dissecting Colistin Resistance Mechanisms in Extensively Drug-Resistant Acinetobacter baumannii Clinical Isolates. MBio (2019) 10(4):e01083-19. doi: 10.1128/mBio.01083-19 31311879 PMC6635527

[132] Jones PW Taylor DM Williams DR Finney M Iorwerth A Webster D. Using wound fluid analyses to identify trace element requirements for efficient healing. J Wound Care (2001) 10(6):205–8. doi: 10.12968/jowc.2001.10.6.26084 12964354

[133] Sheldon JR Skaar EP. Metals as Phagocyte Antimicrobial Effectors. Curr Opin Immunol (2019) 60:1–9. doi: 10.1016/j.coi.2019.04.002 31063946 PMC6800623

[134] Wagner D Maser J Lai B Cai Z Barry CE Höner zu Bentrup K. Elemental Analysis of Mycobacterium Avium-, Mycobacterium Tuberculosis-, and Mycobacterium Smegmatis -Containing Phagosomes Indicates Pathogen-Induced Microenvironments Within the Host Cell’s Endosomal System. J Immunol (2005) 174(3):1491–500. doi: 10.4049/jimmunol.174.3.1491 15661908

[135] Williams CL Neu HM Alamneh YA Reddinger RM Jacobs AC Singh S. Characterisation of Acinetobacter baumannii Copper Resistance Reveals a Role in Virulence. Front Microbiol (2020) 11:16. doi: 10.3389/fmicb.2020.00016 32117089 PMC7015863

[136] Pawar S Ashraf I Lahiri KMMa. Computational Identification of Indispensable Virulence Proteins of Salmonella Typhi Ct18. In: Current Topics in Salmonella and Salmonellosis. InTech (2017). doi: 10.5772/66489

[137] Rajput A Seif Y Choudhary KS Dalldorf C Poudel S Monk JM. Pangenome Analytics Reveal Two-Component Systems as Conserved Targets in ESKAPEE Pathogens. MSystems (2021) 6(1):e00981-20. doi: 10.1128/mSystems.00981-20 33500331 PMC7842365

[138] Kanehisa M. The KEGG Databases at GenomeNet. Nucleic Acids Res (2002) 30(1):42–6. doi: 10.1093/nar/30.1.42 PMC99091 11752249

[139] Chen L Zheng D Liu B Yang J Jin Q. VFDB 2016: Hierarchical and Refined Dataset for Big Data Analysis—10 years on. Nucleic Acids Res (2016) 44(D1):D694–7. doi: 10.1093/nar/gkv1239 PMC4702877 26578559

[140] McArthur AG Waglechner N Nizam F Yan A Azad MA Baylay AJ. The Comprehensive Antibiotic Resistance Database. Antimicrob Agents Chemother (2013) 57(7):3348–57. doi: 10.1128/AAC.00419-13 PMC3697360 23650175

[141] Kaur H Kalia M Taneja N. Identification of Novel Non-homologous Drug Targets Against Acinetobacter baumannii Using Subtractive Genomics and Comparative Metabolic Pathway Analysis. Microb Pathog (2021) 152:104608. doi: 10.1016/j.micpath.2020.104608 33166618

[142] Wishart DS Knox C Guo AC Cheng D Shrivastava S Tzur D. DrugBank: A Knowledgebase for Drugs, Drug Actions and Drug Targets. Nucleic Acids Res (2008) 36(suppl_1):D901–6. doi: 10.1093/nar/gkm958 PMC2238889 18048412

[143] Luo H Lin Y Gao F Zhang C-T Zhang R. DEG 10, An Update of the Database of Essential Genes that Includes both Protein-Coding Genes and Noncoding Genomic Elements: Table 1. Nucleic Acids Res (2014) 42(D1):D574–80. doi: 10.1093/nar/gkt1131 PMC3965060 24243843

[144] Uddin R Masood F Azam SS Wadood A. Identification of Putative non-Host Essential Genes and Novel Drug Targets Against Acinetobacter baumannii by *in Silico* Comparative Genome Analysis. Microbial Pathog (2019) 128:28–35. doi: 10.1016/j.micpath.2018.12.015 30550846

[145] Abdella M Abdella B Lahiri C. Rediscovering and Repurposing Natural Microbial Macromolecules Through Computational Approaches. In. Microbial and Natural Macromolecules (2021) (pp.373–400). Academic Press.

[146] Tillery LM Barrett KF Dranow DM Craig J Shek R Chun I. Toward a Structome of Acinetobacter baumannii Drug Targets. Protein Sci A Publ Protein Soc (2020) 29(3):789–802. doi: 10.1002/pro.3826 PMC7020997 31930600

[147] Neshani A Sedighian H Mirhosseini SA Ghazvini K Zare H Jahangiri A. Antimicrobial Peptides as a Promising Treatment Option Against Acinetobacter baumannii Infections. Microb Pathog. (2020) 146:104238. doi: 10.1016/j.micpath.2020.104238 32387392

[148] Jung C-J Liao Y-D Hsu C-C Huang T-Y Chuang Y-C Chen J-W. Identification of Potential Therapeutic Antimicrobial Peptides Against Acinetobacter baumannii in a Mouse Model of Pneumonia. Sci Rep (2021) 11(1):7318. doi: 10.1038/s41598-021-86844-5 33795739 PMC8016998

[149] Park J Shin E Yeom J-H Choi Y Joo M Lee M. Gold Nanoparticle-DNA Aptamer-Assisted Delivery of Antimicrobial Peptide Effectively Inhibits Acinetobacter baumannii Infection in Mice. J Microbiol (2022) 60(1):128–36. doi: 10.1007/s12275-022-1620-3 34964948

[150] Sacco F Bitossi C Casciaro B Loffredo MR Fabiano G Torrini L. The Antimicrobial Peptide Esc(1-21) Synergizes With Colistin in Inhibiting the Growth and in Killing Multidrug Resistant Acinetobacter baumannii Strains. Antibiotics (2022) 11(2):234. doi: 10.3390/antibiotics11020234 35203836 PMC8868345

[151] He Q Zhao L Li G Shen Y Hu Y Wang Y. The Antimicrobial Cyclic Peptide B2 Combats Multidrug Resistant Acinetobacter baumannii Infection. New J Chem (2022) 46(14):6577–86. doi: 10.1039/D1NJ05353A

[152] Torres MDT Melo MCR Crescenzi O Notomista E de la Fuente-Nunez C. Mining for Encrypted Peptide Antibiotics in the Human Proteome. Nat Biomed Eng (2022) 6(1):67–75. doi: 10.1038/s41551-021-00801-1 34737399

